# Supplementary material for: Probiotics as an Alternative to Antibiotics: Genomic and Physiological Characterization of Aerobic Spore Formers from the Human Intestine
Source: Microorganisms. 2023 Jul 31;11(8):1978. doi: 10.3390/microorganisms11081978 (PMC10458579; doi:10.3390/microorganisms11081978)
Supplement: Supplementary file 1 [file microorganisms-11-01978-s001.zip › microorganisms-2521160-supplementary.pdf]

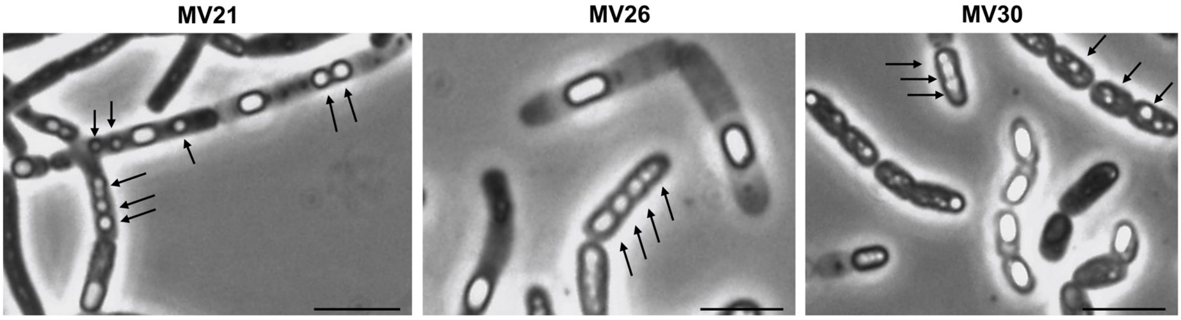

Figure S1: Microscopy analysis of strains producing parasporal structures (arrows)

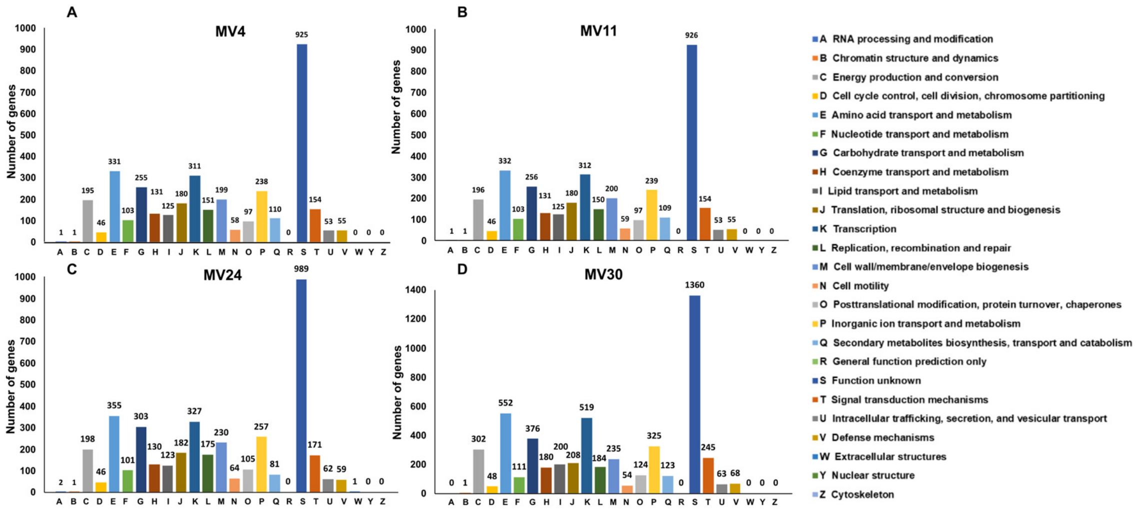

Figure S2: The COG function annotation of *B. velezensis* MV4 (A) and MV11 (B), *B. subtilis* MV24 (C) and *P. megaterium* MV30 (D)

Supplementary Tables

Table S1. Hemolytic activity on blood agar plates.

Strain	Hemolysis	Strain	Hemolysis
MV2	α	MV19	β
MV3	β	MV20	γ
MV4	γ	MV21	α
MV5	α	MV22	α
MV6	γ	MV23	α
MV7	α	MV24	γ
MV8	α	MV25	α
MV9	α	MV26	γ
MV10	α	MV27	α
MV11	γ	MV28	α
MV12	α	MV29	α
MV13	α	MV30	γ
MV14	γ	MV31	α

MV15	γ	MV32	α
MV16	α	MV33	α
MV17	γ		

Table S2. Toxin genes present in the genomes of MV4, MV11, MV24 and MV30

Toxin	Related genes	MV4	MV11	MV24	MV30	*B.cereus* ATCC 14579	*B.clausii* KSM-K16	*B.subtilis subsp. subtilis str.* 168
**Anthrax toxin**	*cya*	-	-	-	-	-	-	-
	lef	-	-	-	-	-	-	-
	pagA	-	-	-	-	-	-	-
**Anthrolysin O/Cereolysin O/Hemolysin I**	*alo*	-	-	-	-	+	-	-
**Cereulide**	*cesA*	-	-	-	-	-	-	-
	cesB	-	-	-	-	-	-	-
	cesC	-	-	-	-	-	-	-
	cesD	-	-	-	-	-	-	-
	cesH	-	-	-	-	-	-	-
	cesP	-	-	-	-	-	-	-
	cesT	-	-	-	-	-	-	-
**Certhrax**	*cer*	-	-	-	-	-	-	-
**Cytotoxin K (Hemolysin IV)**	*cytK*	-	-	-	-	+	-	-
**Hemolysin II**	*hlyII*	-	-	-	-	+	-	-
**Hemolysin III**	*hlyIII*	+	+	+	+	+	+	+
**Hemolytic enterotoxin HBL**	*hblA*	-	-	-	-	+	-	-
	hblC	-	-	-	-	+	-	-
	hblD	-	-	-	-	+	-	-
**Insecticidal crystalline toxins**	*cry*	-	-	-	-	-	-	-
	cyt	-	-	-	-	-	-	-
	vip	-	-	-	-	-	-	-
**Non-hemolytic enterotoxin (Nhe)**	*nheA*	-	-	-	-	+	-	-
	nheB	-	-	-	-	+	-	-
	nheC	-	-	-	-	+	-	-
**Phytotoxin phaseolotoxin (*Pseudomonas*)**	*cysC1*	+	+	-	+	-	-	-
**Cytolysin (*Enterococcus*)**	cylR2	-	-	-	+	-	-	-

Table S3. List of the most similar known clusters of NRPs found in the genomes of MV4, MV11, MV24, MV30.

	Type	Lenght (bp)	Most similar known cluster		Similarity
	Terpene	17265	Unknown		
	transAT-PKS	87827	Macrolactin H	Polyketide	100%
	transAT-PKS,T3PKS,NRPS	100368	Bacillaene	Polyketide+NRP	100%
	NRPS,transAT-PKS,betalactone	107697	Fengycin	NRP	86%
	Terpene	21883	Unknown		
**MV4**	T3PKS	41100	Unknown		
	transAT-PKS	93789	Difficidin	Polyketide	100%
	NRPS,RiPP-like	50498	Bacillibactin	NRP	100%
	Other	41418	Bacilysin	Other	100%
	PKS-like	41244	Butirosin A/butirosin B	Saccharide	7%
	Phosphonate	12141	Unknown		
	NRPS	65011	Surfactin	NRP:Lipopeptide	82%
	NRPS	92475	Fengycin	NRP	20%
	terpene	17265	Unknown		
	transAT-PKS	87827	macrolactin H	Polyketide	100%
	transAT-PKS,T3PKS,NRPS	100368	bacillaene	Polyketide + NRP	100%
	RiPP-like,NRP-metallophore,NRPS	50498	bacillibactin	NRP	100%
	other	41418	bacilysin	Other	100%
	transAT-PKS-like	65218	difficidin	Polyketide + NRP	53%
	T3PKS	41100	Unknown		
	NRPS	43212	surfactin	NRP:Lipopeptide	39%
**MV11**	transAT-PKS	53939	difficidin	Polyketide	46%
	PKS-like	41244	butirosin A/butirosin B	Saccharide	7%
	phosphonate	12141	Unknown		
	NRPS	43033	Unknown		
	NRPS,transAT-PKS,betalactone	107806	fengycin	NRP	86%
	terpene	21883	Unknown		
	NRPS	47196	surfactin	NRP:Lipopeptide	47%
	transAT-PKS-like	63711	difficidin	Polyketide + NRP	26%
	NRPS	95735	fengycin	NRP	20%
	terpene	20391	Unknown		
	NRP-metallophore,NRPS	45583	bacillibactin	NRP	100%
	other	41418	bacilysin	Other	100%
	sactipeptide	21611	subtilosin A	RiPP:Thiopeptide	100%
**MV24**	NRPS-like,betalactone,NRPS	50754	fengycin	NRP	100%
	T3PKS	41097	1-carbapen-2-em-3-carboxylic acid	Other	16%
	terpene	21898	Unknown		
	NRPS	54627	surfactin	NRP:Lipopeptide	78%
	terpene	19989	carotenoid	Terpene	50%
**MV30**	phosphonate	17423			

RRE-containing	20266			
T3PKS	41085			
RiPP-like	11436			
terpene	18349	surfactin	NRP:Lipopeptide	13%
NI-siderophore	16575	synechobactin C9/synechobactin C11/synechobactin 13/synechobactin 14/synechobactin 16/synechobactin A/synechobactin B/synechobactin C	Other	23%
terpene	21868			

Table S4. Matrix-related proteins putatively encoded by the genomes of MV4, MV11, MV24 and MV30.

Protein	Protein found in MV4 (% identity)	Protein found in MV11 (% identity)	Protein found in MV24 (% identity)	Protein found in MV30 (% identity)
**TasA**	MV4_1282 (83.5)	Contig_2_93 (83.5)	MV24_1447 (99.2)	MV30_0609 (37.3)
**SipW**	MV4_1283 (70.0)	Contig_2_94 (70.0)	MV24_1446 (100)	MV30_0424 (29.8)
**TapA**	MV4_1284 (48.2)	Contig_2_95 (48.2)	MV24_1445 (98.4)	MV30_4765 (37.9)
**SinI**	MV4_1280 (71.9)	Contig_2_91 (71.9)	MV24_1449 (100)	MV30_2683 (52.9)
**SinR**	MV4_1281 (97.6)	Contig_2_92 (97.6)	MV24_1448 (100)	MV30_2684 (43.2)
**Spo0A**	MV4_1243 (97.0)	Contig_2_54 (97.0)	MV24_1487 (100)	MV30_1811 (84.3)
**EpsA**	MV4_2261 (65.2)	Contig_3_210 (65.2)	MV24_0888 (98.8)	MV30_0512 (38.5)
**EpsB**	MV4_2262 (85.9)	Contig_3_211 (85.9)	MV24_0889 (99.4)	MV30_0513 (59.3)
**EpsC**	MV4_2263 (82.6)	Contig_3_212 (82.6)	MV24_0890 (99.8)	MV30_5596 (25.2)
**EpsD**	MV4_2264 (74.5)	Contig_3_213 (74.5)	MV24_0891 (99.2)	MV30_0500 (25.4)
**EpsE**	MV4_2265 (83.9)	Contig_3_214 (83.9)	MV24_0892 (100)	MV30_0496 (33.0)
**EpsF**	MV4_2266 (62.5)	Contig_3_215 (62.5)	MV24_0893 (99.4)	MV30_0500 (27.9)
**EpsG**	MV4_2267 (85.9)	Contig_3_216 (85.9)	MV24_0894 (99.0)	MV30_0502 (23.5)
**EpsH**	MV4_2268 (70.2)	Contig_3_217 (70.2)	MV24_0895 (98.9)	MV30_0501 (27.6)
**EpsI**	MV4_2269 (76.0)	Contig_3_218 (76.0)	MV24_0896 (99.7)	MV30_1501 (21.8)
**EpsJ**	MV4_2270 (64.1)	Contig_3_219 (64.1)	MV24_0897 (89.2)	MV30_0501 (36.7)
**EpsK**	MV4_2271 (77.2)	Contig_3_220 (77.2)	MV24_0898 (98.2)	MV30_1993 (22.0)
**EpsL**	MV4_2272(79.4)	Contig_3_221 (78.7)	MV24_0899 (97.0)	MV30_0495 (42.9)
**EpsM**	MV4_2273 (69.2)	Contig_3_222 (69.2)	MV24_0900 (96.6)	MV30_5613 (33.8)
**EpsN**	MV4_2274 (73.2)	Contig_3_223 (73.2)	MV24_0901 (97.8)	MV30_5605 (31.3)
**EpsO**	MV4_2275 (65.2)	Contig_3_224 (65.2)	MV24_0902 (99.6)	MV30_5942 (28.9)
**PurA**	MV4_3474 (97.7)	Contig_4_74 (97.7)	MV24_2773 (99.5)	MV30_4676 (91.1)
**PurB**	MV4_3088 (96.9)	Contig_7_55 (96.9)	MV24_1260 (100)	MV30_4850 (84.7)
**PurC**	MV4_3087 (88.7)	Contig_7_56 (88.7)	MV24_1259 (97.7)	MV30_4849 (59.9)
**PurD**	MV4_3079 (86.1)	Contig_7_64 (86.1)	MV24_1251 (99.7)	MV30_4841 (66.7)
**PurE**	MV4_3090 (88.0)	Contig_7_53 (88.0)	MV24_1262 (98.9)	MV30_4852 (73.9)
**PurF**	MV4_3083 (98.3)	Contig_7_60 (98.3)	MV24_1255 (100)	MV30_4845 (79.4)
**PurH**	MV4_3080 (92.6)	Contig_7_63 (92.6)	MV24_1252 (99.8)	MV30_4842 (73.8)
**PurK**	MV4_3089 (83.0)	Contig_7_54 (83.0)	MV24_1261 (98.2)	MV30_4851 (51.6)
**PurL**	MV4_3084 (96.0)	Contig_7_59 (96.0)	MV24_1256 (99.4)	MV30_4846 (76.6)
**PurM**	MV4_3082 (95.0)	Contig_7_61 (95.0)	MV24_1254 (99.6)	MV30_4844 (73.0)
**PurN**	MV4_3081 (84.6)	Contig_7_62 (84.6)	MV24_1253 (99.5)	MV30_4843 (60.8)
**PurQ**	MV4_3085 (93.0)	Contig_7_58 (93.4)	MV24_1257 (99.1)	MV30_4847 (73.7)
**PurS**	MV4_3086 (100)	Contig_7_57 (100.0)	MV24_1258 (100)	MV30_4848 (92.9)
